# An ecologically motivated image dataset for deep learning yields better models of human vision

**DOI:** 10.1073/pnas.2011417118

**Published:** 2021-02-15

**Authors:** Johannes Mehrer, Courtney J. Spoerer, Emer C. Jones, Nikolaus Kriegeskorte, Tim C. Kietzmann

**Affiliations:** ^a^MRC Cognition and Brain Sciences Unit, University of Cambridge, CB2 7EF Cambridge, United Kingdom;; ^b^Department of Psychology, Zuckerman Institute, Columbia University, New York, NY 10027;; ^c^Donders Institute for Brain, Cognition and Behaviour, Radboud University, 6525 XZ Nijmegen, Netherlands

**Keywords:** human visual system, deep neural networks, computational neuroscience, ecological relevance, computer vision

## Abstract

Deep neural networks provide the current best models of visual information processing in the primate brain. Drawing on work from computer vision, the most commonly used networks are pretrained on data from the ImageNet Large Scale Visual Recognition Challenge. This dataset comprises images from 1,000 categories, selected to provide a challenging testbed for automated visual object recognition systems. Moving beyond this common practice, we here introduce *ecoset*, a collection of >1.5 million images from 565 basic-level categories selected to better capture the distribution of objects relevant to humans. Ecoset categories were chosen to be both frequent in linguistic usage and concrete, thereby mirroring important physical objects in the world. We test the effects of training on this ecologically more valid dataset using multiple instances of two neural network architectures: AlexNet and vNet, a novel architecture designed to mimic the progressive increase in receptive field sizes along the human ventral stream. We show that training on ecoset leads to significant improvements in predicting representations in human higher-level visual cortex and perceptual judgments, surpassing the previous state of the art. Significant and highly consistent benefits are demonstrated for both architectures on two separate functional magnetic resonance imaging (fMRI) datasets and behavioral data, jointly covering responses to 1,292 visual stimuli from a wide variety of object categories. These results suggest that computational visual neuroscience may take better advantage of the deep learning framework by using image sets that reflect the human perceptual and cognitive experience. Ecoset and trained network models are openly available to the research community.

## Significance

Inspired by core principles of information processing in the brain, deep neural networks (DNNs) have demonstrated remarkable success in computer vision applications. At the same time, networks trained on the task of object classification exhibit similarities to representations found in the primate visual system. This result is surprising because the datasets commonly used for training are designed to be engineering challenges. Here, we use linguistic corpus statistics and human concreteness ratings as guiding principles to design a resource that more closely mirrors categories that are relevant to humans. The result is ecoset, a collection of 1.5 million images from 565 basic-level categories. We show that ecoset-trained DNNs yield better models of human higher-level visual cortex and human behavior.

**Fig. 1.**
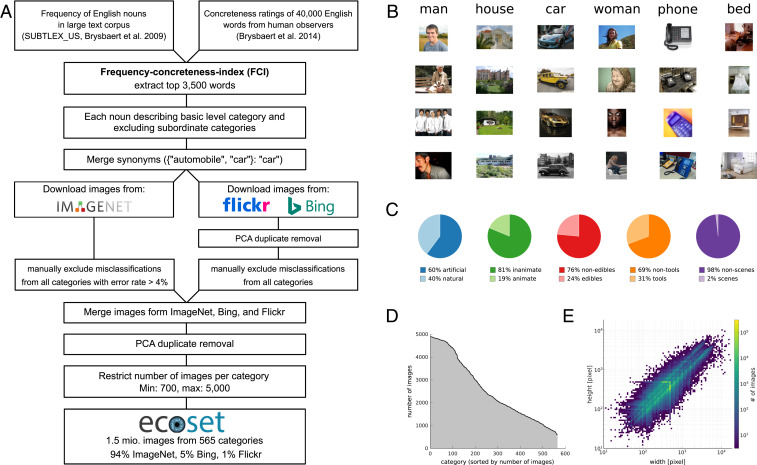
Ecoset overview. (*A*) Flow diagram depicting the steps taken during dataset creation. This includes category selection and curation as well as image processing (search/download, duplicate removal, and label-cleaning procedures). (*B*) Example images from the six categories with FCI (shown in decreasing order from left to right). (*C*) Superordinate category overview. (*D*) Distribution of the number of images per category. (*E*) Distribution of image sizes (log-transformed width and height).

Training deep neural networks (DNNs) end to end on large-scale datasets has led to dramatic advances in computer vision. Computational neuroscience, in turn, found that the representations in these task-trained models exhibit striking similarities to those in the primate visual system (1–3). Although hierarchical convolutional network architectures were inspired by the primate visual system, such similarities are surprising as the images used for network training are selected to serve as a computer vision benchmark. For example, the 1,000 categories to be distinguished in the commonly used 2012 ImageNet Large Scale Visual Recognition Challenge, referred to as ILSVRC 2012 for brevity (4), include 120 different dog breeds but lack categories for humans. In contrast, the human visual system contains multiple regions with a strong preference for human faces and body parts (5). This observation suggests that computational modeling of the human visual system may benefit from novel datasets that more closely mirror the human experience to take full advantage of the deep learning framework (6–9). Here, we introduce *ecoset*, a large-scale image dataset designed for human visual neuroscience, which consists of >1.5 million images from 565 basic-level categories (only 12.7% of ecoset images also appear in ILSVRC 2012). Category selection was based on English nouns that most frequently occur in spoken language [estimated on a set of 51 million words obtained from American television and film subtitles (10)] and concreteness ratings from human observers (11). Ecoset therefore consists of basic-level categories (including human categories man, woman, and child) that describe physical things in the world (rather than abstract concepts) that are important to humans (Fig. 1, see *Materials and Methods* for details on category and image selection procedures). To test whether training DNNs on ecoset rather than ILSVRC 2012 might help to better explain cortical representations in human higher-visual cortex, we train various network instances on both ecoset and ILSVRC 2012 and compare their internal representations against data from two independent functional magnetic resonance imaging (fMRI) studies of human vision (12, 13) as well as human behavioral data (14).

## Results

To quantify the agreement between representations found in DNNs and the brain, we use representational similarity analysis (RSA; 15), which characterizes a system’s population code by means of a representational dissimilarity matrix (RDM, correlation distance). DNNs were shown the same stimuli as human observers (>1,200 images of various object categories), and the resulting network RDMs were compared to RDMs extracted from higher-level visual cortex (HVC) of individual human observers.

A good neural network model of a given brain region should exhibit the same distribution of computational features and thereby predict the representational geometry (16) as captured by the brain RDM. We therefore did not perform any model fitting [i.e., reweighting (1) or linear encoding of the DNN activation profiles (3)], which would enable a model with a different distribution of features to nevertheless perform well (17). The effects of training on ecoset rather than ILSVRC 2012 were tested using two separate network architectures: AlexNet (version 2, 18), one of the most frequently used computer vision networks in computational neuroscience, and vNet, a novel 10-layer convolutional DNN that mimics the progressive increase in foveal receptive field sizes along multiple areas of the human ventral stream (V1, V2, V3, hV4, LO, TO, pFUS, and mFUS; see *Materials and Methods*) as previously estimated by population receptive field mapping (19, 20). While computer vision networks, engineered for task performance, exist in large variety and complexity, testing ecoset on biologically more realistic models brings both the architecture and training set into closer alignment with the task of modeling brain function. Such networks thereby constitute a more rigorous test for the effects of changing the training data. To account for individual differences among DNNs (21), 10 network instances per architecture, each initialized with different random weights, were trained on each dataset (see *Materials and Methods*).

**Fig. 2.**
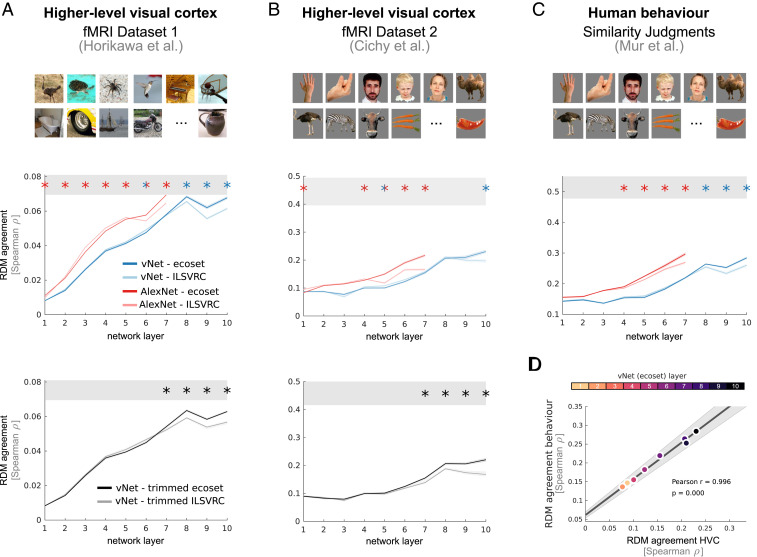
Training on ecoset rather than ILSVRC 2012 improves the alignment between DNN representations and human HVC as well as with human perceptual similarity judgments. (*A*) Data for fMRI dataset 1. (*A*, middle row) Benefits of training on ecoset were true for both architectures tested (AlexNet, shown in red, as well as vNet, shown in blue). Lower bound of the noise ceiling shown as the lower edge of the gray bar, stars indicate significant differences at *P* < 0.01, Bonferroni corrected for the number of network layers. To estimate statistical significance, each network instance of a given architecture was correlated with data from each human participant. To summarize the performance of a network instance, the average match across all human individuals was computed. Based on these data, permutation tests were performed comparing network instances trained on either ecoset or ILSVRC. Error bars indicate 95% CI across network instances (see *Materials and Methods* for further details). (*A*, bottom row) Benefits of training on ecoset persist when controlling for the number of images and the number of categories in the two training datasets. (*B*) Effects obtained for fMRI dataset 1 replicate in a separate fMRI dataset (dataset 2). (*C*) DNNs trained on ecoset also exhibit better alignment with human perceptual similarity judgments (behavioral dataset, ecoset-trained network shown in black, ILSVRC 2012 in gray). (*D*) The model fit between HVC and human behavior exhibits a strong positive relationship (data for various vNet network layers shown as data points).

Analyses of the learned network features via RSA revealed significant benefits in predicting human higher-level visual representations when training on ecoset rather than ILSVRC 2012. This was true for both architectures and both fMRI datasets tested (Fig. 2 *A* and *B* and *SI Appendix*, Fig. S1). For fMRI dataset 1 (12), which comprises cortical responses to 1,200 natural scenes recorded from each of five human participants, later networks layers exhibited the best match to HVC. This is in line with the literature, which commonly relies on these layers for modeling higher-level visual computations (1, 12, 22). When training AlexNet with ecoset, we found layers six and seven to be more similar to human HVC than their ILSVRC-trained counterparts (permutation test, *P* < 0.01, Bonferroni corrected for the number of network layers; see *Materials and Methods* for details, please note the effect reversal observed in earlier layers, all of which, however, provide an overall worse model of HVC). Despite no parameter fitting, the predictive power of layer seven of ecoset-trained AlexNet was on par with human observers (matching the lower bound of the noise ceiling, i.e., the predictive performance of the grand average computed over all other participants). Similar effects were observed for vNet, which exhibits significantly higher alignment with HVC representations when trained on ecoset in layers eight to 10 (permutation test, *P* < 0.01, Bonferroni corrected; peak similarity at layer eight, 98.3% of the lower bound of the noise ceiling). In the final network layers, ecoset training led to an increase of up to 13 percentage points in the explained proportion of explainable variance (the latter estimated as the lower bound of the noise ceiling) for AlexNet and 17 percentage points for vNet (the total variance explained increased by 15% for AlexNet and up to 21% for vNet).

FMRI dataset 2 (13) consists of cortical responses to 92 objects from a diverse set of categories shown against a gray background, recorded from each of 15 human participants. Testing against these data revealed that layers five to seven of ecoset-trained AlexNet more closely mirrored HVC representations (permutation test, *P* < 0.01, Bonferroni corrected, Fig. 2*B*, middle row). For vNet, significant benefits for ecoset training were observed in layer 10 (permutation test, *P* < 0.01, Bonferroni corrected). Layer 10 of ecoset-trained vNet performed at 59.3% of the lower bound of the noise ceiling (i.e., the predictive performance of the average of 14 held out participants) and layer seven of AlexNet performed at 54.8%. In the final network layers, ecoset training led to an increase of up to 12 percentage points in the explained proportion of explainable variance for AlexNet and 9 percentage points for vNet (the total variance explained increased by 70% for AlexNet and 37% for vNet). Together, the benefits of training with ecoset, as observed for both architectures and both datasets, are consistent with the interpretation that the visuo-semantic representations of human HVC in part reflect the distribution of categories in human language (see *SI Appendix*, Fig. S2 for results on early visual cortex [V1-V3] and visual areas V4/LO1-3, while benefits of ecoset training generalize to visual areas of intermediate complexity, no coherent difference in performance across training sets was observed for early visual areas).

To exclude explanations based on dataset differences in the number of categories and number of images per category, we created “trimmed” versions of both ecoset and ILSVRC 2012 while controlling for these factors. We then trained 10 vNet instances on each and compared their internal representations analogous to the original analyses. Replicating our previous results in this more conservative control, we observed significant benefits of training vNet with ecoset compared to ILSVRC 2012 in layers seven to 10 for both fMRI datasets 1 and 2 (Fig. 2 *A* and *B*, bottom row, all *P* < 0.05, Bonferroni corrected).

**Fig. 3.**
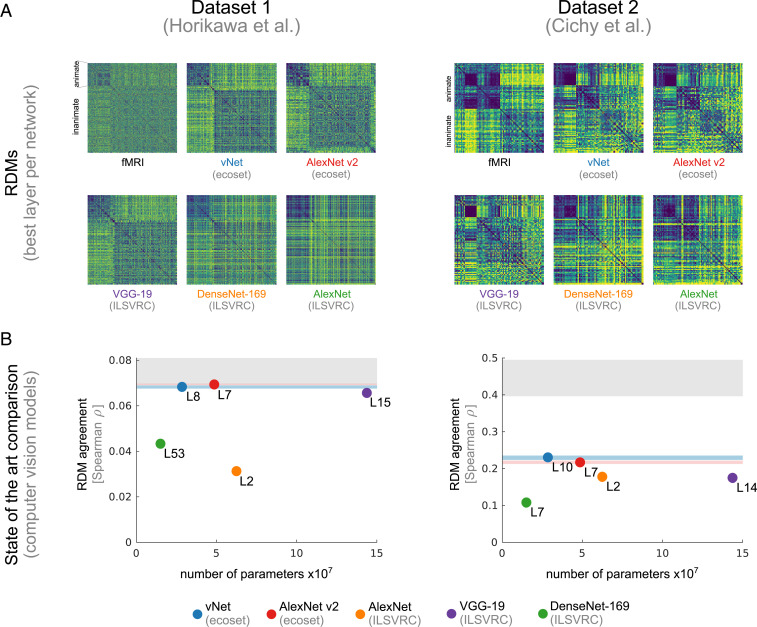
Comparing ecoset-trained DNNs to the state of the art. (*A*) Target RDMs from human HVC shown together with RDMs extracted from various deep neural network models (best layer selected for each with dataset 1 on the left and dataset 2 on the right). (*B*) Agreement with human HVC plotted against model parametric complexity. vNet and AlexNet v2, both trained on ecoset, significantly outperform state of the art DNN models pretrained on ILSVRC 2012 (DenseNet-169, VGG-19, and the original AlexNet). Error bars shown in blue and red indicate 95% CI.

Next, we compared our ecoset-trained networks (AlexNet v2 and vNet) against high-performance, large-scale computer vision DNNs that represent the state of the art in computational neuroscience (1–3). These included the original, pretrained AlexNet (23), VGG-19 (24), and DenseNet-169 (25). Compared to vNet (10 layers, 28 M parameters), VGG-19 and DenseNet-169 are deeper, and AlexNet and VGG-19 have substantially more parameters (61 M for the original AlexNet and 144 M for VGG-19 because of their fully connected layers, which contain 96% and 86% of parameters for the original AlexNet and VGG-19, respectively). For each DNN, we selected the layer that best predicted the fMRI data for further analyses (all possible layers used as candidates for AlexNet and VGG-19 and all concatenation layers for DenseNet-169). We find that ecoset-trained vNet and ecoset-trained AlexNet v2 significantly outperformed all tested pretrained computer vision models in terms of predicting human HVC representations (*P* < 0.05, bootstrapped CIs, Fig. 3). This was true for both fMRI datasets tested.

To better understand why ecoset-trained DNNs perform better in predicting human HVC, we separately compared the representational dissimilarities of each experimental stimulus to all other experimental stimuli across DNN models and brain data [i.e., we performed the previous analyses on each column of the respective RDM separately instead of on the whole RDM at once (26)]. Focusing on the final layers of AlexNet and vNet, which had previously shown clear improvements for both fMRI datasets, we found significant predictive advantages for animate objects (human and animal) over inanimate objects (manmade and natural objects). That is, ecoset training resulted in better alignment of the representational dissimilarities for animate objects, including the relations among animate objects as well as the relations to inanimate objects. This effect was highly consistent across both network architectures and datasets (permutation tests of the interaction effect, testing whether the benefits of ecoset training were larger for predictions of representational dissimilarities of animate rather than inanimate objects, all *P* < 0.01; see *SI Appendix*, Fig. S3 and *Supplementary Text* for details). These results indicate that ecoset training may yield more brain-like representations for animate object categories as well as their relation to inanimate objects, mirroring large-scale organizational principles found in the human ventral stream (26, 27). Interestingly, this effect arises despite ILSVRC having a higher percentage of animate objects (39% in ILSVRC versus 19% in ecoset). This raises the possibility that the advantage of using ecoset originates from a more appropriate selection of object categories as well as from the requirement for object categorization on the basic level instead of a larger set of subordinate category distinctions.

Expanding our previous analyses of cortical representations in human HVC to behavior, we tested whether ecoset training also yields network internal representations that more closely mirror human perceptual judgments. We used behavioral data obtained via inverse multidimensional scaling (inverse MDS), a task in which participants perform multiple two-dimensional (2D) arrangements of real-world objects to indicate their perceived similarity (14, 28). The stimulus set used in this experiment was equivalent to the stimuli from fMRI dataset 2. For each participant, inverse MDS results in a perceptual RDM of equivalent format to the previously analyzed dissimilarity matrices. Mirroring our previous analysis approach, no model fitting was performed to align DNN and behavioral data. Ecoset-trained networks significantly outperformed ILSVRC-trained network instances in their alignment with human perceptual judgments (Fig. 2*C*). This was true for both AlexNet (significant benefits in layers four to seven, permutation test, *P* < 0.05, Bonferroni corrected; peak similarity at layer seven, 62.1% of the lower bound of the noise ceiling) and vNet (significant benefits in layers eight to 10, permutation test, *P* < 0.01, Bonferroni corrected; peak similarity at layer 10, 59.5% of the lower bound of the noise ceiling). In the final network layer, ecoset training led to an increase of 6.8 percentage points in the explained proportion of explainable variance for AlexNet and 5.7 percentage points for vNet (the total variance explained increased by 21% for AlexNet and 19% for vNet). Significant benefits in later network layers were also observed for vNets trained on the trimmed ILSVRC and ecoset datasets, which control the number of images and categories (*SI Appendix*, Fig. S4). Moreover, in line with previous reports of alignment between perceptual judgments and human inferior temporal cortex (14), we find a significant correlation between our networks’ ability to mirror human HVC and human perceptual judgments [vNet rho = 0.996; AlexNet rho = 0.987; both *P* < 0.001; robust Pearson correlation (29); Fig. 2*D*].

Finally, the human visual system contains multiple higher-level visual regions in which neurons exhibit selectivity for images of faces (5). To test in how far face selectivity is mirrored in our network models, we searched for face-selective units by running in silico electrophysiology experiments in which we contrast the units' responsiveness to images showing either faces or places and computed the percentage of units with significant face selectivity for each network layer and network instance (see *Materials and Methods*). Although faces are not a separate object category, we reasoned that the existence of related ecoset categories, such as woman, man, and child, would naturally lead to the more prominent emergence of face-selective units in higher-level network representations. Indeed, we observe that the deepest network layers of both architectures exhibit both the highest percentage of face-selective units as well as a significant increase when training on ecoset rather than ILSVRC (Wilcoxon signed-rank test across network instances, *P* < 0.05, Bonferroni corrected for the number of layers per network, *SI Appendix*, Fig. S5). For AlexNet, the average percentage of face-selective units increased from 9.3 to 12.2%, and for vNet, we observed an increase from 4.3 to 6.9%.

## Conclusions

Ecoset provides an alternative to ILSVRC 2012 by featuring a more ecologically valid distribution of categories based on spoken word frequency and human concreteness ratings. We have shown that training deep neural networks on ecoset, instead of the commonly used data from ILSVRC, produces DNN representational spaces that are not only more consistent with those found in human HVC but that also align better with human perceptual judgments. The size of these statistically significant benefits is modest, but as we have shown here, they replicate across two architectures, trained instances of these architectures, two brain-activity data sets, and human similarity judgments. Moreover, the observed benefits for predicting representational geometries of animate objects (including response similarities among animate objects as well as between animate and inanimate objects) was consistent across network architectures and datasets.

As a step in the direction of increasing the biological plausibility of deep network architectures, we here designed vNet such that the model receptive field sizes mirror the progression of foveal receptive field sizes across the human visual hierarchy. Future work should explore in how far the interplay of ecoset and the introduction of further biological details, such as recurrence (30–34), skip connections, and more biologically more realistic learning rules can further improve model predictions (6, 8). Another aspect worth considering is the learning objective. We here trained all DNNs to optimize for categorization performance. While this task is undoubtedly of ecological relevance, the explanatory power of unsupervised objectives (35–37), semantically better-informed training targets, and their interplay with ecoset will be worth considering going forward.

To test our networks against brain data, we here focused on similarities between representations learned by DNNs and the ones found in human HVC across two separate and diverse fMRI datasets. Whereas dataset 1 focused on stimulus variety (1,200 natural scenes shown to each participant), dataset 2 relied on high repetition rates for fewer stimuli (92 segmented objects). We think that this dataset diversity is an important aspect for evaluating new computational resources, such as ecoset and vNet. Although we observed significant benefits of training on ecoset in all cases, it should be noted that the lower bound of the noise ceiling of dataset 1 in particular is comparably low, likely due to individual differences among the small number of participants and because of our choice of using single-trial responses to individual images rather than averaging images showing the same object categories. The resulting variability in lower-bound estimates needs to be taken into account when interpreting the observed high network performance. Individual differences also exist among DNNs (21), and it will be of interest to relate these two phenomena.

In addition to acquiring better fMRI datasets to further underline the generality of the effects observed [more data per subject, more stimulus variety including diverse object poses and orientations (38), higher field, higher contrast-to-noise ratio], a promising avenue of future research is large-scale, in silico neurophysiology, which could be used to better understand how unit selectivity changes as a result of training with ecologically more valid input statistics. Here, we presented a first foray into this domain by showing that ecoset training leads to an increase in face-selective units in final network layers. Moving further into the domain of behavior, it will be of interest to perform in-depth tests of ecoset-trained networks (supervised or unsupervised) to compare their task performance and error distributions against human behavioral data (39–42).

To enable rapid adoption by the community, ecoset is openly available for research purposes at https://dx.doi.org/10.24433/CO.4784989.v1. We also provide all trained vNet and AlexNet v2 instances along with a web interface that allows users to extract activation patterns and RDMs in response to their own stimulus sets. In addition to use cases in computational neuroscience, we expect ecoset to be useful to the machine learning community where it provides a challenging computer vision benchmark.

## Materials and Methods

### Ecoset Dataset.

#### Overview.

This section provides an in-depth description of ecoset category and image selection procedures. Please refer to *SI Appendix*, Table S1 for a list of all 565 ecoset categories together with their word frequency, concreteness rating, frequency concreteness index (FCI), and the corresponding number of images.

Ecoset was created as a large-scale image resource for deep learning and human visual neuroscience more generally (see ref. 43 for a related dataset designed for experimental work in psychology and neuroscience). A total of 565 categories were selected based on the following: 1) their word frequency in American television and film subtitles (SUBTLEX_US, 10), 2) the perceived concreteness by human observers (11), and 3) the availability of a minimum of 700 images. Images were sourced via the overall ImageNet database (the same resource used for ILSVRC 2012) or obtained under CC BY-NC-SA 2.0 license from Bing image search and Flickr. Thorough data cleaning procedures were put in place to remove duplicates and to assure an expected misclassification rate per category of <4%.

#### Category selection.

The aim of ecoset was to provide the community with a dataset that contains ecologically more valid categories than typical computer vision datasets that were designed toward engineering goals. Starting from all nouns in the English language, two parameters were used to guide the selection process. First, the frequency at which a given noun occurs in a linguistic corpus of spoken language was used as a proxy for concept importance. Second, human ratings of each noun's concreteness were used to focus on categories that have a physical realization and which can therefore be readily visualized (compare for example the nouns “strawberry” and “hope,” which are at opposing ends of the concreteness spectrum). Only nouns with an associated concreteness rating of 4.0 or higher were considered for inclusion. We then combined the two selection parameters, frequency, and concreteness by defining an FCI (defined below). This enabled us to focus on the most common, most concrete nouns of the English language.

Estimates of noun frequency were based on a linguistic corpus consisting of American television and film subtitles (SUBTLEX_US, 10). Concreteness estimates were publicly available (11). These data were collected via Amazon Mechanical Turk, asking participants to rate words (40,000 total) with regard to their concreteness on a five-level Likert scale. Frequency estimates and concreteness ratings were each standardized to a range between 0 and 1. FCI was subsequently defined as the average standardized frequency and concreteness. It ranges from 0 to 1. We computed the FCI for all words contained in the concreteness rating dataset (11) and processed the 3,500 nouns with the highest FCI rating in depth.

$$FCI=0.5*\frac{wordf\;requency}{\text{max}\left(word\;frequency\right)}+0.5*\frac{concreteness}{\max\left(concreteness\right)}$$

Only nouns that describe basic-level categories were considered for inclusion. Please note that the definition of basic-level categories is a matter of an ongoing scientific debate, and basic-level judgments can vary across individuals (44). Because of its inherently subjective nature, the classification of nouns that constitute basic-level categories was performed repeatedly across the whole set by the authors, and the selection was subsequently verified by two project independent researchers.

In detail, category selection was performed using the following criteria: First, nouns describing subordinate and superordinate categories were excluded in favor of basic-level categories (for example, “terrier” and “animal” were excluded in favor of “dog”). Moreover, only single-word concepts were included as candidates, excluding separated compound nouns as their own entities (e.g., “sail boat,” “fire truck,” etc.), as these are often part of a basic-level category (in the previous example “boat” and “truck,” respectively). Third, we excluded nouns describing object parts (e.g., “wheel,” “roof,” or “hand”), as they constitute parts of objects in other basic-level categories, thereby rendering the image categories ambiguous. Moreover, although the human brain exhibits visual areas that appear uniquely selective to certain categories, such as body parts [faces, hands, etc. (5)], such selectivity should ideally emerge as a result of network training according to an externally defined objective. Including them as explicit training targets would prohibit analyses of such emergent phenomena. Fourth, synonyms were combined into a single category (e.g., “automobile” and “car” are summarized into a single “car” category). The resulting set of nouns describes basic-level categories for which the resulting images can be ascribed to a single category as commonly used in many one-hot encoded deep learning applications. The final set of ecoset categories is distinctively different from the category selection ILSVRC. First, ecoset focuses on basic-level categories rather than category labels from various levels of categorical abstraction. Second, only 24% of categories in ecoset have a matching ILSVRC category. As a more conservative estimate, we furthermore included comparisons across category levels by including all WordNet hyponyms of each ecoset category for comparisons (e.g., counting the ILSVRC category “Brittany spaniel” as a match to ecoset’s “dog”). Please note that this match across category levels (i.e., matching basic-level ecoset categories to subordinate categories in ILSVRC) is quite conservative, as the underlying categorization task is different. Nevertheless, we find only 16% of ecoset categories to have a matching WordNet hyponym in ILSVRC.

#### Image selection and technical validation.

Most images (∼94%) were sourced from the ImageNet database [of which the well-known ILSVRC 2012 dataset with its 1,000 object categories is a subset (4)]. To compute the actual image-based overlap between ecoset and ILSVRC, we ran a similar analysis used for duplicate removals, as described in detail below, across both datasets (ecoset and ILSVRC). We find that only 12.7% of images in ecoset also appear in ILSVRC 2012, indicating little overlap between the two datasets. To find images matching a given ecoset category, we used the ImageNet web interface to manually search for appropriate WordNet synsets to be included. Multiple synsets could be selected as sources for a given category.

As additional resources for finding images, we used Bing and Flickr image searches based on the category names, synonyms, and their translations into other languages (French, Spanish, Italian, and German). Image search via Flickr and Bing was constrained to images under CC BY-NC-SA 2.0 license. For the Flickr application programming interface (API), we chose option one (NonCommercial-ShareAlike License), and for the Bing API we chose the option “share,” both referring to CC BY-NC-SA 2.0. In the final ecoset dataset, 5.1% of images were obtained via Bing and 1.4% were obtained via Flickr.

To maximize the probability that all images in the ecoset dataset are unique, a duplicate removal procedure was implemented. This was designed to not only spot exact duplicates but also more subtle variations, including different sizes or different aspect ratios. Duplicate removal was performed for each category separately. First, we cropped the center square of all images of the category, resized them to 128 × 128 pixels, and performed a principal component analysis (PCA) preserving 90% of the variance across all images of that category. The similarity of all image pairs was computed based on a Pearson correlation between their respective PCA component loadings. Based on 10 exemplary categories, we established a cutoff value above which a pair of images was labeled as duplicate (Pearson *r* > 0.975). If multiple duplicates per category instance existed, only the image with the largest resolution was kept for ecoset.

We performed a manual image inspection procedure to ensure that the ecoset images were correctly classified. All images sourced via Bing and Flickr (97,379 images in total) were visually inspected, and misclassified instances were removed. For images obtained via ImageNet, we visually inspected 100 randomly sampled instances from each ecoset category. If more than four of those 100 images were found to be misclassifications, the whole category was manually cleaned. Otherwise, all images were included. As a result of this cleaning procedure, we expect the error rate of all ecoset categories to be lower than 4%.

Due to the large-scale sampling of images via the web required for ecoset, some of the images used to train the DNN models contained nudity. These images were removed in creating the publicly available version of ecoset to allow for more straight forward adoption by all community members. Images were marked for removal if the probability of containing not safe for work (NSFW) material exceeded 0.8, estimated using a DNN trained for NSFW detection [Yahoo (45), https://github.com/yahoo/open_nsfw]. Note that only 118 (out of >1.5 million) images had to be removed.

#### Trimmed dataset versions.

Ecoset and ILSVRC 2012 differ in the number of categories (565 versus 1,000) and in the distribution of the number of images per category. These differences might confound their ability to predict neural data. To control for this possibility, we created “trimmed” versions of both datasets that are identical in the number of categories and the distribution of the number of images per category. For this, we selected all 565 categories from ecoset and a subset of 565 randomly chosen categories from ILSVRC 2012. To hold the number of images per category equal across trimmed image sets, while retaining the maximally possible number of images, the following procedure was implemented. First, we ordered the 565 categories of ecoset and trimmed ILSVRC 2012 according to category size and paired the categories from the sorted list across images sets (e.g., pairing the largest category of ecoset with the largest category of ILSVRC). For each category pair, one from each dataset, we then selected the larger category and randomly removed images to match the number of images in the smaller category. As a result, trimmed ecoset and trimmed ILSVRC both contain 565 categories and follow the same distribution of category sizes with minimally 600 to maximally 1,300 images per category in the respective training sets.

#### Limitations of ecological validity.

As stated above, the category selection of ecoset was based on human concreteness ratings and word frequencies in a corpus consisting of American television and film subtitles. This undoubtedly biases the category selection toward Western cultures. Image inclusion was based on the availability via Bing/Flickr search results as well as the existence of relevant ImageNet categories. Images depicting people, specifically the categories “man,” “woman,” and “child,” were not sampled according to census distributions (age, ethnicity, gender, etc.). Moreover, ecoset image and category distributions do not reflect the naturalistic, egocentric visual input typically encountered in the everyday life of infant and adults (46, 47).

### Deep Neural Network Architectures.

#### vNet.

**Fig. 4.**
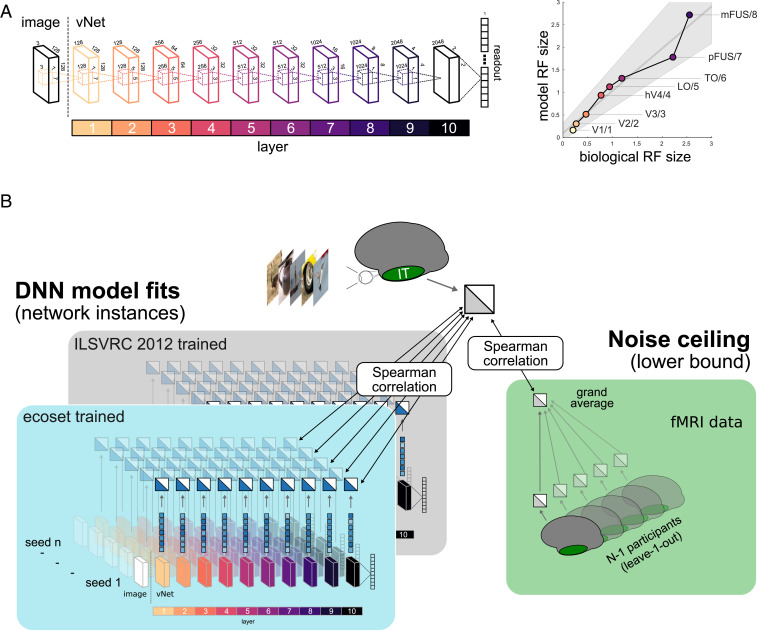
vNet design and statistical procedures. (*A*) The vNet architecture was designed such that the effective kernel sizes across its layers approximate the progressive increase in average RF sizes in the central 3° of visual angle along human ventral stream areas. (*B*) To compare the representations learned by DNNs and the ones found in human HVC, all network instances were shown the same stimuli as the human observers to extract their activation patterns. Based on these patterns, RDMs were computed, one per layer and network instance. These dissimilarity matrices were then compared to the HVC RDMs of each individual participant using Spearman’s correlation. We used the average of the individual participant correlations to estimate the predictive performance of a given network instance and layer (see section *Statistical Comparisons between Human IT and DNN Representations* for details). The data noise ceiling was computed by comparing individual participant RDMs to the average RDM of all remaining participants, again using a Spearman’s correlation.

The vNet architecture, as introduced here, was designed such that the effective kernel sizes across network layers mirror the progressive increase of average receptive field (RF) sizes along multiple areas of the human ventral stream (Fig. 4*A*). As model targets, we chose human V1, V2, V3, hV4, LO, TO, pFUS, and mFUS. As a substantial part of the human ventral stream lies anterior to these eight regions, including object- and concept-selective regions (48–50), we included two more layers to the final network while following the same incremental trends in RF size. The network’s total field of view was set to 3° of visual angle, and the human receptive field sizes were defined based on population receptive field estimates obtained at an eccentricity of 0.75° visual angle to mirror the average foveal RF size (19, 20). Each vNet layer consists of a convolution operation, dropout, max pooling, group norm, and a ReLU nonlinearity (no max pooling for the input and layers [1, 2, 5, and 6]). Each of 10 network instances per training set was trained for 80 epochs using Adam as optimizer, group normalization, a minibatch size of 256, and dropout with a probability of 0.2. The networks reached an average top-one test performance of 65.3% for ecoset and 59.3% for ILSVRC 2012. A weighted loss was used to correct for dataset imbalances in the number of images across object categories.

#### AlexNet v2 (retrained).

To compare the effects of training on ecoset versus ILSVRC 2012 on more commonly used deep neural network architecture, we retrained AlexNet in its 2014 refined version (18). The most important difference of this version, apart from slightly different numbers of feature maps in the first two layers, is the use of data instead of model parallelization. All training hyperparameters were chosen as closely as possible to the original publication (learning rate 0.01, dropout 0.5, minibatch size 128, momentum 0.9, and weight decay 0.0005). Varying the random seed for the initial network weights, we trained 10 network instances each on both datasets. Networks were trained for 90 epochs. The retrained AlexNet instances reached an average top-one accuracy of 63.8% on ecoset and 58.1% on ILSVRC 2012. A weighted loss was used to correct for dataset imbalances in the number of images across object categories.

#### Off-the-shelf computer vision networks (original AlexNet, Densenet, VGG19).

In addition to training refined AlexNet (v2) and vNet instances on ecoset and ILSVRC 2012, we tested other commonly used network architectures from the domain of computer vision for their ability to mirror representations in human HVC. These included VGG19 (24), DenseNet-169 (25), and the original 2012 AlexNet architecture (23). DenseNet and VGG19 were obtained via Keras applications. Original AlexNet was obtained via the Caffe model zoo (“bvlc_alexnet”).

#### DNN RDM extraction.

To compare the network internal representations to those observed in human ventral stream areas, we presented the networks with the same two stimulus sets that were presented to the human participants in the imaging experiments. We then computed layer-based network RDMs for each instance by calculating all pairwise distances between the high-dimensional network responses (using correlation distance as for the fMRI data).

#### DNN in silico electrophysiology.

We estimated the percentage of face-selective cells in each layer of vNet and Alexnet by contrasting, for each network layer, the units’ responsiveness to images showing either faces or places (50 stimuli each, taken from the ecoset test set). Units were deemed face selective if they exhibited a significantly higher response to faces than places (Wilcoxon rank-sum test *P* < 0.05, false discovery rate (FDR) corrected across all units across the whole network). For each network layer, we then test for significant differences in the percentage of face-selective units, using network instances as observations (results Bonferroni corrected for the number of layers per network at *P* < 0.05).

### fMRI Data.

#### Overview.

Data from human early visual cortex (EVC) and HVC were obtained from two fMRI datasets (12, 13). Acquisition and preprocessing details can be found in the corresponding publications. RSA was used to characterize human ventral stream representations in both regions of interest (ROIs). RDMs were computed for each participant and ROI using correlation distance.

To estimate observation noise in the respective fMRI dataset and ROI, the RDM of each participant was individually compared to the grand average RDM of the remaining participants. The average of these correlations is equivalent to the lower bound of the noise ceiling (1).

#### Dataset 1.

This dataset consists of data from five healthy participants presented with a set of 1,200 photographs of natural objects with natural background. Stimuli were presented at 12° of visual angle (12). A total of 312 stimuli contain animate objects (eight humans) and 888 inanimate objects (64 plants). See Fig. 2 (left column) for exemplary images from this set. Low-level visual cortex was defined to include areas V1 to V3. HVC was manually delineated on the flattened surface of the individual participants to include the lateral occipital complex (LOC), fusiform face area (FFA), and parahippocampal place area (PPA).

#### Dataset 2.

This dataset consists of data from 15 healthy participants who were presented photographs of 92 objects shown against a gray background (see Fig. 2, right column for examples). Stimuli were presented at 2.9° visual angle (13). The 92 images were sampled from human (12) and nonhuman faces (12) and bodies (12 and 12 each) as well as natural and manmade inanimate objects (23 and 21 images, respectively). EVC included areas V1 to V3, as defined in the Glasser atlas (51). HVC was defined to include regions along the IT and parahippocampal cortex, as defined in (30). For both ROIs, EVC and HVC, the 500 most visually responsive voxels were selected for subsequent analyses.

### Statistical Comparisons between Human IT and DNN Representations.

The following procedure was implemented to test whether training on ecoset rather than ILSVRC 2012 leads to network internal representations that more closely mirror the ones found in early and high-level regions of the human ventral stream. For each dataset, we extracted brain RDMs from each participant and ROI as well as DNN-based RDMs for each network architecture, instance, and layer. For each ROI and dataset, we then iterated through all participants and correlated the upper triangle of the corresponding brain RDM with the network RDMs using the Spearman’s rank correlation coefficient (see Fig. 4*B* for a graphical depiction of the analysis pipeline for a single participant). As a summary statistic, we averaged the correlation values from all participants for each network instance and layer. This value describes the average RDM similarity of a given network instance and layer with all human participants. To test whether training on ecoset rather than ILSVRC 2012 led to significant differences in model alignment with representations in human IT, we took the RDM correlations obtained for each model instance and performed a permutation test in which we shuffled the dataset labels across network instances (10,000 iterations for vNet and all possible 252 permutations for AlexNet v2). The test was performed for each network layer, architecture, and fMRI dataset separately. To control the family-wise error rate, we used a Bonferroni correction for the number of network layers (i.e., the number of tests performed for each network architecture, ROI, and dataset; vNet: 10 and AlexNet v2: seven). Moreover, we estimated the 95% CIs for the predictive performance of both architectures using bootstrapping of the network instances (1,000 samples).

### Human Behavioral Data.

In addition to testing our models against representations found in human HVC, we compared our network internal representations for their agreement with behavioral data, obtained from human similarity judgments (14, 28). Using the same stimulus set as in fMRI dataset 2, participants were asked to communicate perceptual object similarity by arranging sets of multiple object images in 2D on a computer screen by mouse drag and drop. Combining data from multiple trials of this arrangement task, a perceptual similarity matrix can be computed for each of 16 participants. This matrix has the same format as the RDMs used previously. Statistical comparisons between perceptual and DNN dissimilarity matrices were performed in analogy to the previous fMRI analyses. The data were previously presented in ref. 14.

## Supplementary Material

Supplementary File

## Data Availability

All materials presented in this paper (ecoset dataset, pretrained networks, and test stimuli) are openly available for research purposes via CodeOcean (52): https://dx.doi.org/10.24433/CO.4784989.v1.
