# Supplementary material for: An ecologically motivated image dataset for deep learning yields better models of human vision
Source: Proc Natl Acad Sci U S A. 2021 Feb 15;118(8):e2011417118. doi: 10.1073/pnas.2011417118 (PMC7923360; doi:10.1073/pnas.2011417118)
Supplement: Supplementary File [file pnas.2011417118.sapp.pdf]

PNAS
www.pnas.org

Supplementary Information for

Ecoset: an ecologically more valid visual diet for deep learning yields better models of human higher-level visual cortex and behavior

Johannes Mehrer[a], Courtney J Spoerer[a], Emer C Jones[a], Nikolaus Kriegeskorte[b], & Tim C Kietzmann[a,c,*]

Paste corresponding author name here
Email: t.kietzmann@donders.ru.nl

This PDF file includes:

Figure S1-S5
Supplementary text: Category-specific RDM analysis
Table S1

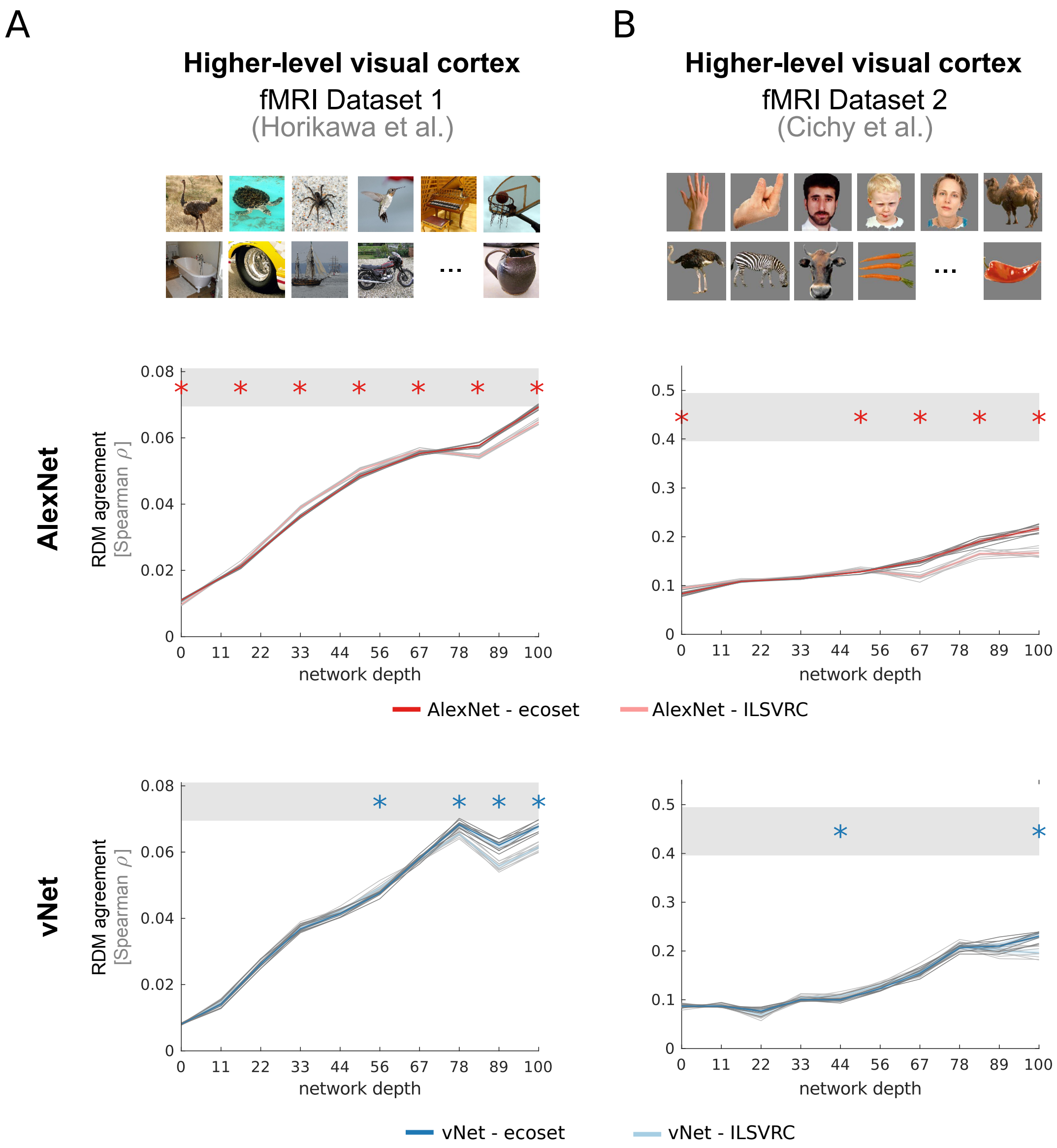

Fig. S1 | Training on ecoset rather than ILSVRC 2012 improves the alignment between DNN representations and human HVC. Same data as in Figure 2 of the main manuscript, results for dataset 1 shown left, dataset 2 shown right. We here show the performance of individual network instances and focus, instead of on layers, on relative network depth. AlexNet and vNet results shown separately to increase visibility.

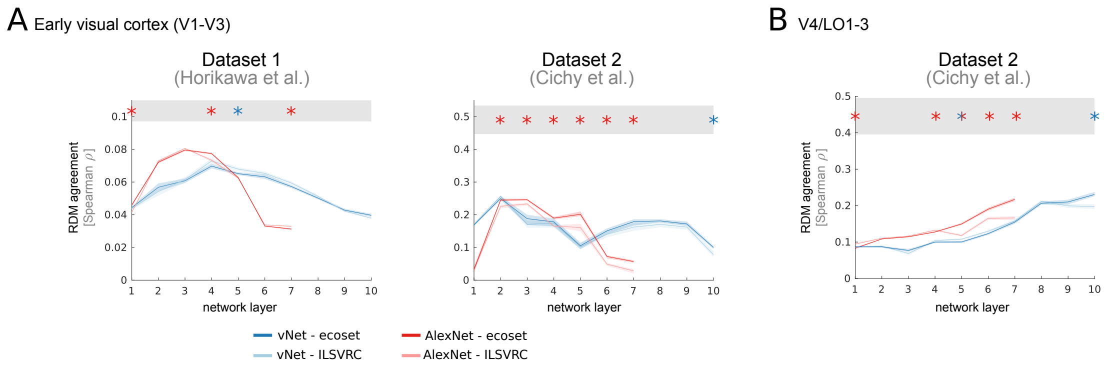

Fig. S2 | Early and intermediate visual cortex analysis. Although the design of ecoset was focused on a more appropriate higher-level category selection, we also compared ecoset- and ILSVRC-trained network instances against representations found in early visual cortex (EVC) and mid-level visual areas (V4/LO1-3). *(A)* In contrast to HVC, which is best explained by later network layers in both architectures (shown in Figure 2 of the main paper), EVC was best predicted by earlier network layers (dataset 1: layer 3 for AlexNet, layer 4 for vNet; dataset 2: layer 2 for ecoset-trained AlexNet and vNet). In contrast to the coherent benefits of training on ecoset for HVC, the results for EVC were less consistent across datasets (stars indicate $p<0.05$, permutation test, Bonferroni corrected). *(B)* For mid-level visual areas V4/LO1-3, we observe similar effects to HVC with later network layers exhibiting the best RDM agreement, as well as consistent and significant benefits resulting from ecoset training.

Supplementary text: Category-specific RDM analysis

To better understand why ecoset trained networks exhibit improved alignment with representations in HVC, we extended our previous analyses by running them in an image-specific way. For this, we separately Spearman correlated each column of the brain RDMs with the corresponding column of the DNN RDMs. This column-based analysis tests the agreement between the representational distances of a given stimulus to the set of all other stimuli. As a summary statistic, we averaged all correlation coefficients of stimuli belonging to a given superordinate category (animate/inanimate), and subsequently to subcategories within the superordinate ones (human/animal within animate, and natural/manmade for inanimates). We then tested whether ecoset-trained DNNs yield higher overall correlations for stimuli of a given category by performing a permutation test in which we shuffle the dataset labels across network instances (10,000 iterations for vNet and all possible 252 permutations for AlexNet v2). To control the family-wise error rate, we used a Bonferroni correction for the number tests performed per network and dataset. This analysis is inspired by Kriegeskorte et al. 2008 (see Figure S2), please note that the diagonal of the RDMs was excluded as it is zero by definition.

To test whether the effects of ecoset training were larger for animate rather than inanimate objects, we computed the effect size of ecoset training for each pair of networks (each pair initialized with the same random seed, but one trained on ecoset, the other one on ILSVRC) for stimuli showing animate and inanimate objects, respectively. This provided us with an effect size estimate for each network pair, and superordinate object category (animate/inanimate), enabling us to statistically test for an interaction effect. The latter was performed using a permutation test, as detailed above.

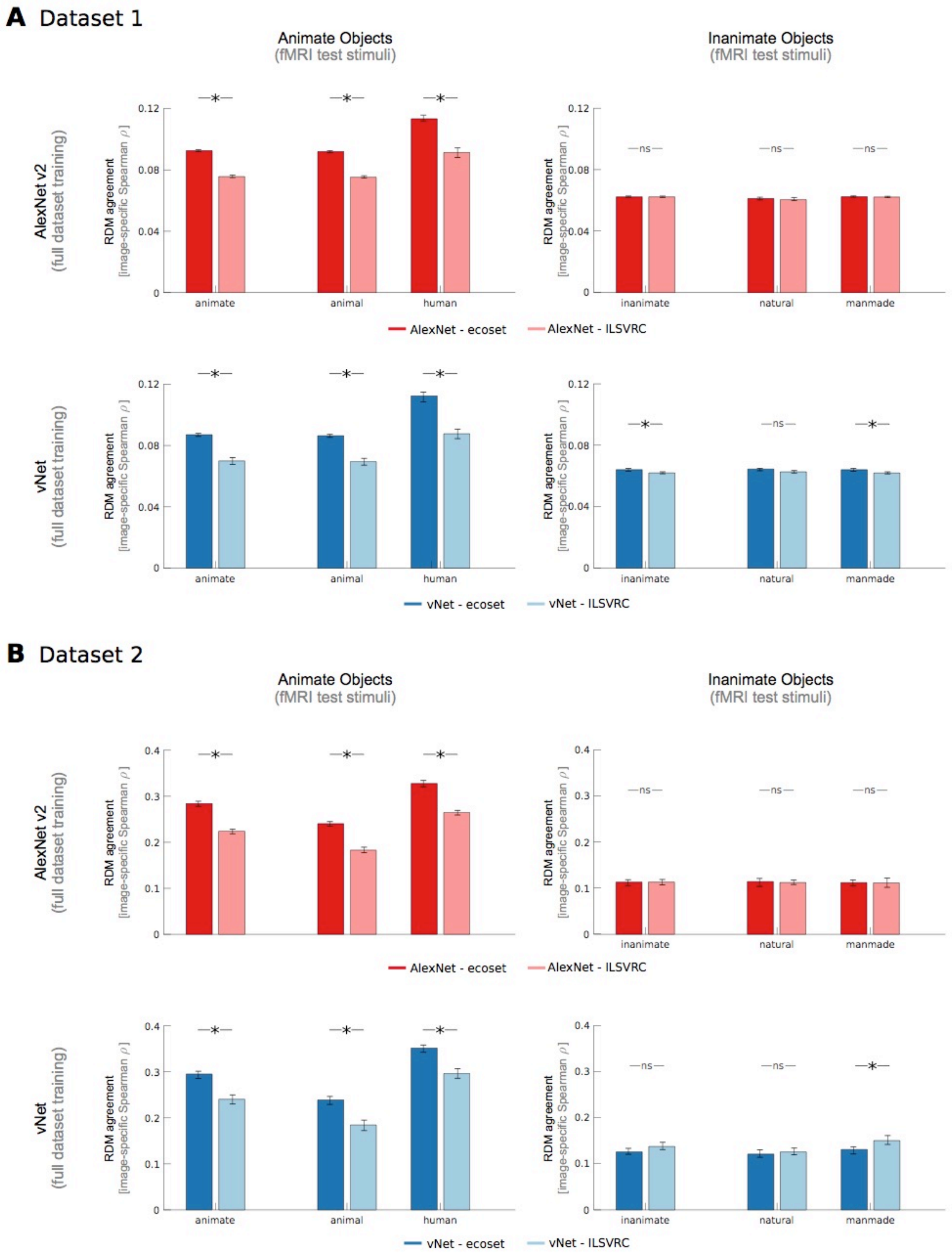

Fig. S3 | Category-specific RDM analysis. *(A)* We tested several superordinate object categories for the effect of network training on ecoset rather than ILSVRC. Instead of correlating whole RDMs, as in the main analysis, we instead computed the Spearman correlation coefficient for each RDM column separately (image-specific RDM analysis).

We then averaged the coefficients for all stimuli belonging to a given superordinate category. This analysis revealed that the benefits of training on ecoset are strongest for animate objects (including the relations among themselves, as well as to inanimate objects). This was true for both network architectures and datasets tested (results for Dataset 2 shown in panel *(B)*.

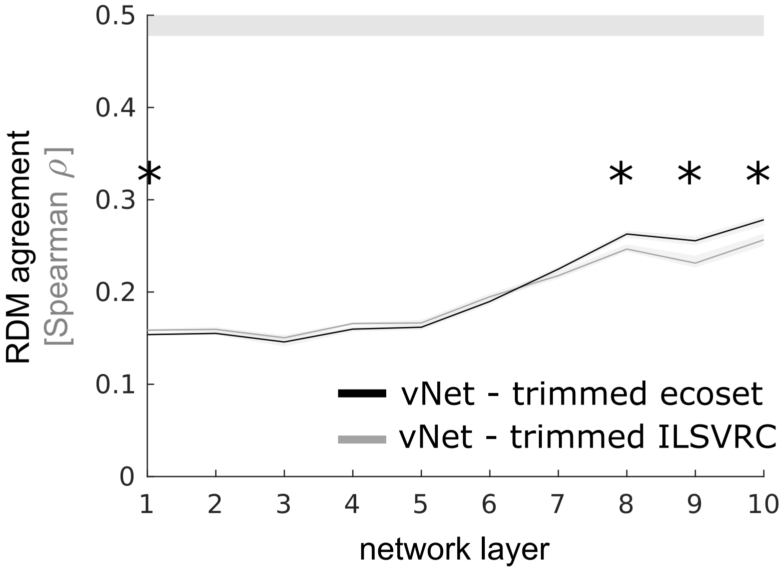

Fig. S4 | DNN alignment with perceptual similarity judgments. To ensure that the benefits of ecoset training are not due dataset differences in the number of categories or images, we trained two sets of networks on trimmed versions of ILSVRC and ecoset, respectively, and compared the network RDMs with RDMs obtained via perceptual similarity judgments. Consistent with our main results, we observe, in higher-level network layers, significantly better alignment between DNNs trained on trimmed ecoset (shown in black) than DNNs trained on trimmed ILSVRC (shown in grey, permutation test, $p<0.01$, Bonferroni corrected for the number of network layers).

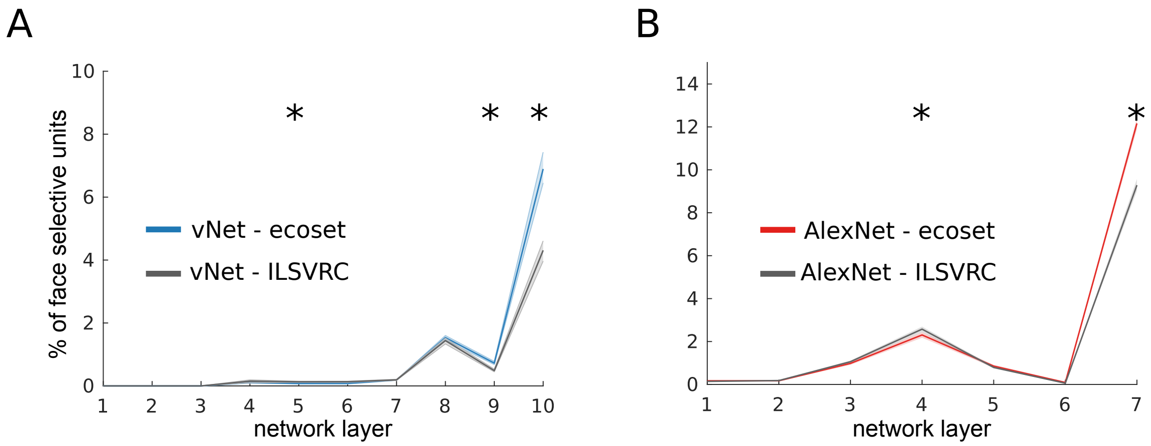

Fig. S5 | Face-selectivity analysis. We contrasted the percentage of face-selective units when training neural networks on ecoset or ILSVRC. Consistently for both network architectures (vNet shown in *(A)*, AlexNet shown in *(B)*), we find a substantial increase in face selective units in the final network layers when training with ecoset. Please note that there also seems to be a marginal reduction in face-selective units in mid-level layers. Stars indicate significant differences (Wilcoxon signed-rank test, $p<0.05$, Bonferroni corrected for the number of network layers).

Table S1 | Ecoset categories

category index	category name	word frequency	concreteness	FCI	# of images
001	man	94133	4.79	0.979000	4620
002	house	26214	5.00	0.639239	4905
003	car	24636	4.89	0.619857	4988
004	woman	22166	4.46	0.563738	4898
005	phone	13756	4.86	0.559067	4732
006	bed	9543	5.00	0.550689	4821
007	gun	10873	4.83	0.540753	4862
008	book	9026	4.90	0.537943	4928
009	dog	9835	4.85	0.537240	4904
010	ball	5353	5.00	0.528433	3640
011	fire	10990	4.68	0.526375	4957
012	horse	4737	5.00	0.525161	4997
013	city	8624	4.79	0.524808	1416
014	fish	4258	5.00	0.522617	1392
015	child	8040	4.78	0.520706	4391
016	boat	4885	4.93	0.518947	1464
017	table	5387	4.90	0.518614	4983
018	tree	3315	5.00	0.517608	2471
019	clock	2990	5.00	0.515882	4942
020	bag	4796	4.90	0.515475	4981
021	camera	2907	5.00	0.515441	4948
022	cup	2634	5.00	0.513991	4781
023	ship	5043	4.87	0.513787	4955
024	kitchen	2974	4.97	0.512797	1954
025	key	4430	4.89	0.512531	3713
026	bird	2318	5.00	0.512312	3325
027	pig	1996	5.00	0.510602	4304
028	bus	3783	4.90	0.510094	4053
029	bridge	2331	4.97	0.509381	4836
030	pizza	1709	5.00	0.509078	4870
031	computer	3011	4.93	0.508993	4591
032	church	3553	4.90	0.508872	4921
033	doll	1263	5.00	0.506709	3880
034	bell	2006	4.96	0.506655	4868
035	stairs	1212	5.00	0.506438	2081
036	apple	1207	5.00	0.506411	3542
037	flower	1161	5.00	0.506167	4550
038	ring	4730	4.81	0.506124	1792
039	snake	1140	5.00	0.506055	4906
040	mountain	1805	4.96	0.505587	4996
041	road	5709	4.75	0.505324	1597
042	wall	3605	4.86	0.505148	4837
043	tiger	945	5.00	0.505019	4887
044	toilet	1474	4.97	0.504829	3733
045	train	4848	4.79	0.504751	4768
046	bottle	2588	4.91	0.504747	4510
047	turtle	869	5.00	0.504616	4933
048	cookie	852	5.00	0.504526	950
049	egg	1328	4.97	0.504054	3781
050	river	2829	4.89	0.504027	2532
051	cat	3383	4.86	0.503969	4985
052	truck	3716	4.84	0.503738	4976
053	basket	672	5.00	0.503569	3490
054	bear	2928	4.88	0.503552	3670
055	moon	2548	4.90	0.503534	853
056	milk	2169	4.92	0.503521	2586
057	blanket	662	5.00	0.503516	1806
058	lemon	613	5.00	0.503256	2656

Continuation of Table					
category index	category name	word frequency	concreteness	FCI	# of images
059	frog	603	5.00	0.503203	3597
060	pillow	581	5.00	0.503086	1988
061	elephant	580	5.00	0.503081	4869
062	cow	1301	4.96	0.502910	4834
063	banana	547	5.00	0.502905	3656
064	goat	537	5.00	0.502852	4782
065	knife	2387	4.90	0.502679	4737
066	ladder	472	5.00	0.502507	865
067	popcorn	465	5.00	0.502470	2136
068	refrigerator	427	5.00	0.502268	3476
069	jar	424	5.00	0.502252	3026
070	jail	3602	4.83	0.502133	2199
071	hamburger	397	5.00	0.502109	3693
072	toast	1707	4.93	0.502067	1907
073	umbrella	382	5.00	0.502029	1955
074	bean	349	5.00	0.501854	4741
075	castle	1099	4.96	0.501837	1379
076	flashlight	302	5.00	0.501604	3807
077	tomato	301	5.00	0.501599	4800
078	strawberry	282	5.00	0.501498	2405
079	leopard	276	5.00	0.501466	4290
080	donkey	273	5.00	0.501450	3350
081	axe	249	5.00	0.501323	4381
082	mailbox	212	5.00	0.501126	2892
083	grape	204	5.00	0.501084	4845
084	vase	196	5.00	0.501041	2799
085	carrot	195	5.00	0.501036	2316
086	tractor	190	5.00	0.501009	2452
087	cupcake	167	5.00	0.500887	1070
088	fern	163	5.00	0.500866	4941
089	bagel	156	5.00	0.500829	1832
090	telescope	150	5.00	0.500797	4516
091	cactus	148	5.00	0.500786	4571
092	tent	892	4.96	0.500738	4858
093	microscope	129	5.00	0.500685	4390
094	kite	117	5.00	0.500621	1272
095	lantern	103	5.00	0.500547	2010
096	octopus	99	5.00	0.500526	1054
097	lamp	657	4.97	0.500490	3300
098	blender	85	5.00	0.500451	1295
099	burrito	84	5.00	0.500446	2256
100	mango	84	5.00	0.500446	1874
101	binoculars	80	5.00	0.500425	1704
102	steak	828	4.96	0.500398	2257
103	gravel	73	5.00	0.500388	1456
104	escalator	66	5.00	0.500351	746
105	walrus	57	5.00	0.500303	1179
106	horseshoe	52	5.00	0.500276	1355
107	antelope	50	5.00	0.500266	4428
108	tongs	40	5.00	0.500212	2123
109	porcupine	33	5.00	0.500175	1646
110	camcorder	32	5.00	0.500170	1312
111	mousetrap	30	5.00	0.500159	843
112	lion	783	4.96	0.500159	4644
113	cauliflower	28	5.00	0.500149	2395
114	shower	2097	4.89	0.500138	1877
115	hotdog	20	5.00	0.500106	3407
116	warthog	16	5.00	0.500085	1622
117	thimble	14	5.00	0.500074	1052

Continuation of Table					
category index	category name	word frequency	concreteness	FCI	# of images
118	guardrail	14	5.00	0.500074	1012
119	dustpan	13	5.00	0.500069	1293
120	crawfish	13	5.00	0.500069	1881
121	eyedropper	7	5.00	0.500037	932
122	nectarine	3	5.00	0.500016	1574
123	flyswatter	2	5.00	0.500011	979
124	lollypop	2	5.00	0.500011	919
125	lightbulb	0	5.00	0.500000	2071
126	corn	725	4.96	0.499851	4212
127	cave	713	4.96	0.499787	4537
128	pie	1466	4.92	0.499787	900
129	spider	515	4.97	0.499735	4934
130	bread	1445	4.92	0.499675	4802
131	motorcycle	455	4.97	0.499417	3890
132	monkey	1709	4.90	0.499078	4664
133	whale	574	4.96	0.499049	4895
134	airplane	557	4.96	0.498959	4965
135	shovel	349	4.97	0.498854	3087
136	bucket	511	4.96	0.498714	2308
137	rabbit	1068	4.93	0.498673	2958
138	necklace	497	4.96	0.498640	2386
139	moose	282	4.97	0.498498	1688
140	glass	3096	4.82	0.498445	4841
141	drum	432	4.96	0.498295	4921
142	mop	211	4.97	0.498121	2698
143	bracelet	398	4.96	0.498114	3404
144	spoon	388	4.96	0.498061	3095
145	stove	387	4.96	0.498056	4722
146	lettuce	173	4.97	0.497919	4551
147	ashtray	166	4.97	0.497882	1367
148	lake	1836	4.88	0.497752	1435
149	noodles	309	4.96	0.497641	3890
150	walnut	100	4.97	0.497531	1512
151	pastry	98	4.97	0.497521	2348
152	ferret	83	4.97	0.497441	1929
153	fig	62	4.97	0.497329	1991
154	eggplant	56	4.97	0.497297	1971
155	violin	242	4.96	0.497285	4690
156	chipmunk	42	4.97	0.497223	2813
157	milkshake	42	4.97	0.497223	901
158	blackberry	38	4.97	0.497202	1397
159	sushi	222	4.96	0.497179	1744
160	apricot	32	4.97	0.497170	1666
161	drawers	218	4.96	0.497158	4411
162	doughnut	215	4.96	0.497142	1658
163	lawnmower	24	4.97	0.497127	4465
164	snowplow	20	4.97	0.497106	1092
165	chalkboard	12	4.97	0.497064	1931
166	backpack	186	4.96	0.496988	2622
167	alligator	178	4.96	0.496945	3190
168	cockroach	174	4.96	0.496924	3172
169	lime	168	4.96	0.496892	1681
170	lighthouse	157	4.96	0.496834	2693
171	dolphin	141	4.96	0.496749	4853
172	piano	1268	4.90	0.496735	4825
173	earring	138	4.96	0.496733	2561
174	chicken	3148	4.80	0.496721	4825
175	blueberry	131	4.96	0.496696	3318
176	grapefruit	126	4.96	0.496669	1544

Continuation of Table					
category index	category name	word frequency	concreteness	FCI	# of images
177	thermometer	112	4.96	0.496595	3814
178	cranberry	99	4.96	0.496526	2724
179	iceberg	92	4.96	0.496489	1165
180	wasp	73	4.96	0.496388	3671
181	tweezers	52	4.96	0.496276	1339
182	asparagus	50	4.96	0.496266	2430
183	croissant	45	4.96	0.496239	1602
184	teapot	44	4.96	0.496234	2164
185	needle	608	4.93	0.496229	1854
186	acorn	37	4.96	0.496197	1258
187	bumblebee	33	4.96	0.496175	1727
188	anvil	32	4.96	0.496170	971
189	seashell	16	4.96	0.496085	4887
190	birdhouse	13	4.96	0.496069	1691
191	thyme	10	4.96	0.496053	4256
192	thumbtack	9	4.96	0.496048	1107
193	saltshaker	6	4.96	0.496032	1466
194	paperclip	2	4.96	0.496011	1209
195	matchstick	1	4.96	0.496005	897
196	envelope	513	4.93	0.495725	3787
197	pineapple	130	4.94	0.494691	2079
198	wheelchair	316	4.93	0.494678	2417
199	butterfly	281	4.93	0.494493	4978
200	camel	256	4.93	0.494360	4588
201	balloon	442	4.92	0.494348	3606
202	beach	2888	4.79	0.494340	2583
203	guitar	795	4.90	0.494223	4853
204	crate	209	4.93	0.494110	1187
205	wrench	202	4.93	0.494073	3080
206	eggroll	1	4.94	0.494005	1318
207	taco	158	4.93	0.493839	1183
208	rat	1663	4.85	0.493833	3956
209	meatball	132	4.93	0.493701	2795
210	emerald	131	4.93	0.493696	1386
211	omelet	121	4.93	0.493643	2878
212	sheep	685	4.90	0.493638	4807
213	jukebox	116	4.93	0.493616	1289
214	desert	1427	4.86	0.493580	720
215	raspberry	96	4.93	0.493510	3568
216	snail	90	4.93	0.493478	4763
217	pistachio	77	4.93	0.493409	1443
218	groundhog	76	4.93	0.493404	3521
219	videotape	264	4.92	0.493402	868
220	jellyfish	74	4.93	0.493393	2674
221	carousel	73	4.93	0.493388	2049
222	hippo	72	4.93	0.493382	2001
223	pear	68	4.93	0.493361	3832
224	bullet	1950	4.83	0.493358	1654
225	pliers	59	4.93	0.493313	2990
226	toothpick	52	4.93	0.493276	1142
227	stagecoach	51	4.93	0.493271	1180
228	hourglass	49	4.93	0.493260	1612
229	coliseum	44	4.93	0.493234	2104
230	blowtorch	43	4.93	0.493228	1288
231	treadmill	42	4.93	0.493223	2506
232	cake	2298	4.81	0.493206	3865
233	bobcat	20	4.93	0.493106	1571
234	cumin	16	4.93	0.493085	1162
235	hedgehog	15	4.93	0.493080	1730

Continuation of Table					
category index	category name	word frequency	concreteness	FCI	# of images
236	opossum	4	4.93	0.493021	2426
237	pumpkin	553	4.90	0.492937	3535
238	bowl	1094	4.87	0.492811	4230
239	worm	516	4.90	0.492741	4550
240	candy	1825	4.83	0.492694	4245
241	clarinet	80	4.92	0.492425	2326
242	peanut	630	4.89	0.492346	1656
243	gondola	37	4.92	0.492197	1418
244	padlock	35	4.92	0.492186	2539
245	television	1729	4.83	0.492184	4796
246	barnacle	32	4.92	0.492170	2376
247	leek	15	4.92	0.492080	964
248	cashew	11	4.92	0.492058	2063
249	streetlamp	2	4.92	0.492011	1830
250	razor	351	4.90	0.491864	3552
251	peach	324	4.90	0.491721	2081
252	pudding	314	4.90	0.491668	3726
253	coin	497	4.89	0.491640	1377
254	grasshopper	47	4.91	0.491250	4815
255	pea	199	4.90	0.491057	4033
256	toaster	198	4.90	0.491052	1566
257	chalk	183	4.90	0.490972	858
258	bee	528	4.88	0.490805	4848
259	bicycle	337	4.89	0.490790	4945
260	screwdriver	128	4.90	0.490680	1803
261	pencil	503	4.88	0.490672	3492
262	jaguar	121	4.90	0.490643	1545
263	garlic	306	4.89	0.490625	1767
264	rhino	106	4.90	0.490563	4615
265	wheat	293	4.89	0.490556	1462
266	waterfall	95	4.90	0.490505	1012
267	squirrel	279	4.89	0.490482	4939
268	bulldozer	66	4.90	0.490351	1996
269	closet	1381	4.83	0.490335	2283
270	broom	243	4.89	0.490291	3561
271	starfish	38	4.90	0.490202	2019
272	ladle	38	4.90	0.490202	2481
273	scoreboard	31	4.90	0.490165	1652
274	rice	769	4.86	0.490085	2906
275	crouton	13	4.90	0.490069	971
276	lasagna	183	4.89	0.489972	2352
277	flea	169	4.89	0.489898	893
278	towel	722	4.86	0.489835	4729
279	bench	493	4.87	0.489619	4923
280	hammock	71	4.89	0.489377	757
281	windmill	65	4.89	0.489345	3637
282	pan	627	4.86	0.489330	2966
283	avocado	62	4.89	0.489329	2121
284	guacamole	56	4.89	0.489297	1726
285	cane	425	4.87	0.489257	2022
286	sprinkler	47	4.89	0.489250	2132
287	microphone	232	4.88	0.489232	4475
288	outhouse	38	4.89	0.489202	1494
289	anthill	29	4.89	0.489154	1030
290	tortilla	16	4.89	0.489085	1493
291	eggbeater	12	4.89	0.489064	1378
292	hare	195	4.88	0.489036	3177
293	breadbox	6	4.89	0.489032	1043
294	nest	566	4.86	0.489006	701

Continuation of Table					
category index	category name	word frequency	concreteness	FCI	# of images
295	rifle	743	4.85	0.488947	4842
296	skunk	166	4.88	0.488882	1810
297	barrel	542	4.86	0.488879	4761
298	typewriter	161	4.88	0.488855	4482
299	bun	147	4.88	0.488781	3991
300	hay	325	4.87	0.488726	2687
301	mosquito	93	4.88	0.488494	3694
302	coffin	461	4.86	0.488449	2078
303	newspaper	1208	4.82	0.488416	4797
304	condom	263	4.87	0.488397	702
305	deer	444	4.86	0.488358	4911
306	mouse	975	4.83	0.488179	4515
307	candle	409	4.86	0.488172	3296
308	kumquat	24	4.88	0.488127	2201
309	cymbals	24	4.88	0.488127	995
310	mall	964	4.83	0.488120	1478
311	potato	576	4.85	0.488060	1211
312	candelabra	6	4.88	0.488032	3342
313	knot	188	4.87	0.487999	3799
314	lobster	374	4.86	0.487987	3966
315	crib	316	4.86	0.487678	2503
316	broccoli	116	4.87	0.487616	2416
317	fireworks	287	4.86	0.487524	1507
318	ant	273	4.86	0.487450	4857
319	crowbar	66	4.87	0.487351	703
320	thermostat	58	4.87	0.487308	934
321	caterpillar	57	4.87	0.487303	4617
322	papaya	56	4.87	0.487297	1075
323	ceiling	426	4.85	0.487263	2274
324	zucchini	49	4.87	0.48726	1453
325	pecan	48	4.87	0.487255	1000
326	wallet	1163	4.81	0.487177	2164
327	radish	31	4.87	0.487165	2947
328	onion	216	4.86	0.487147	3939
329	crayon	21	4.87	0.487112	1572
330	pancake	202	4.86	0.487073	1165
331	canoe	182	4.86	0.486967	3873
332	casket	162	4.86	0.486860	1820
333	tunnel	912	4.82	0.486844	872
334	scissors	341	4.85	0.486811	4815
335	cork	146	4.86	0.486775	1936
336	tofu	137	4.86	0.486728	1276
337	zebra	128	4.86	0.486680	4449
338	kangaroo	118	4.86	0.486627	4034
339	hamster	109	4.86	0.486579	3071
340	missile	670	4.83	0.486559	2593
341	dishwasher	103	4.86	0.486547	1025
342	bamboo	80	4.86	0.486425	2549
343	altar	259	4.85	0.486376	3340
344	otter	69	4.86	0.486367	4661
345	nacho	67	4.86	0.486356	1267
346	calculator	66	4.86	0.486351	4195
347	fence	819	4.82	0.486350	4975
348	rhubarb	55	4.86	0.486292	1974
349	stethoscope	48	4.86	0.486255	1516
350	library	1170	4.80	0.486215	3303
351	tadpole	30	4.86	0.486159	1432
352	dollhouse	22	4.86	0.486117	1894
353	trashcan	19	4.86	0.486101	1607

Continuation of Table					
category index	category name	word frequency	concreteness	FCI	# of images
354	guava	13	4.86	0.486069	2038
355	pomegranate	13	4.86	0.486069	2657
356	tamale	12	4.86	0.486064	1389
357	anteater	11	4.86	0.486058	3981
358	plum	174	4.85	0.485924	1486
359	oyster	156	4.85	0.485829	2797
360	cinnamon	152	4.85	0.485807	2086
361	bandage	146	4.85	0.485775	3133
362	elevator	1245	4.79	0.485613	1066
363	mistletoe	98	4.85	0.485521	2274
364	marshmallow	77	4.85	0.485409	1056
365	custard	64	4.85	0.485340	3760
366	wristwatch	59	4.85	0.485313	1660
367	corkscrew	57	4.85	0.485303	1512
368	kazoo	31	4.85	0.485165	904
369	kebab	31	4.85	0.485165	1758
370	granola	30	4.85	0.485159	948
371	gargoyle	27	4.85	0.485143	1911
372	scone	25	4.85	0.485133	1867
373	mantis	23	4.85	0.485122	2641
374	parsnip	4	4.85	0.485021	856
375	curtain	525	4.82	0.484789	2418
376	scorpion	136	4.84	0.484722	1300
377	crown	698	4.81	0.484708	2206
378	lemonade	281	4.83	0.484493	1505
379	wolf	1034	4.79	0.484492	3966
380	bugle	88	4.84	0.484467	4817
381	graveyard	272	4.83	0.484445	2622
382	tumbleweed	22	4.84	0.484117	1302
383	plate	1308	4.77	0.483948	2173
384	dragonfly	145	4.83	0.483770	2111
385	flag	892	4.79	0.483738	1598
386	crocodile	115	4.83	0.483611	3261
387	mushroom	109	4.83	0.483579	4923
388	beetle	105	4.83	0.483558	4961
389	cucumber	101	4.83	0.483536	1741
390	sloth	74	4.83	0.483393	2738
391	dough	810	4.79	0.483302	2307
392	sphinx	52	4.83	0.483276	1864
393	canyon	418	4.81	0.483220	2102
394	iguana	40	4.83	0.483212	3018
395	chalice	32	4.83	0.483170	1524
396	doormat	28	4.83	0.483149	1110
397	hairpin	18	4.83	0.483096	2134
398	aloe	15	4.83	0.483080	4531
399	scaffolding	12	4.83	0.483064	1726
400	platypus	3	4.83	0.483016	1022
401	brownie	180	4.82	0.482956	825
402	casino	1039	4.77	0.482519	1527
403	shrimp	444	4.80	0.482358	3364
404	grate	52	4.82	0.482276	2412
405	loudspeaker	47	4.82	0.482250	4906
406	tower	1165	4.76	0.482188	4899
407	submarine	362	4.80	0.481923	2891
408	rug	531	4.79	0.481820	4677
409	ape	493	4.79	0.481619	2516
410	banner	302	4.80	0.481604	1273
411	syringe	99	4.81	0.481526	2228
412	hanger	69	4.81	0.481367	2212

Continuation of Table					
category index	category name	word frequency	concreteness	FCI	# of images
413	cannon	444	4.79	0.481358	3642
414	kale	28	4.81	0.481149	2205
415	pothole	25	4.81	0.481133	964
416	chili	382	4.79	0.481029	2160
417	waterspout	5	4.81	0.481027	890
418	stadium	312	4.79	0.480657	3653
419	spacecraft	115	4.80	0.480611	1597
420	boar	111	4.80	0.480590	2068
421	cheese	1991	4.70	0.480575	4826
422	celery	95	4.80	0.480505	1215
423	hammer	636	4.77	0.480378	3694
424	matchbook	51	4.80	0.480271	1234
425	coconut	234	4.79	0.480243	2438
426	beet	14	4.80	0.480074	3154
427	stegosaurus	5	4.80	0.480027	1010
428	chocolate	1499	4.72	0.479962	3949
429	muffin	297	4.78	0.479578	3628
430	turnip	88	4.79	0.479467	2058
431	wire	1403	4.72	0.479452	3476
432	chandelier	72	4.79	0.479382	1968
433	forklift	49	4.79	0.479260	1789
434	fondue	47	4.79	0.479250	3425
435	gazebo	46	4.79	0.479244	1864
436	wheelbarrow	31	4.79	0.479165	1645
437	melon	218	4.78	0.479158	4512
438	earpiece	27	4.79	0.479143	2420
439	paintbrush	27	4.79	0.479143	1300
440	bib	25	4.79	0.479133	2013
441	strongbox	16	4.79	0.479085	3333
442	steamroller	12	4.79	0.479064	1310
443	breadfruit	6	4.79	0.479032	1542
444	dishrag	5	4.79	0.479027	1649
445	anchor	378	4.77	0.479008	1945
446	fountain	352	4.77	0.478870	2613
447	parachute	162	4.78	0.478860	3052
448	sink	863	4.74	0.478584	3101
449	radiator	103	4.78	0.478547	2782
450	burner	93	4.78	0.478494	2292
451	llama	72	4.78	0.478382	4258
452	playground	260	4.77	0.478381	3848
453	okra	26	4.78	0.478138	2786
454	gramophone	16	4.78	0.478085	2846
455	burlap	11	4.78	0.478058	2451
456	earwig	5	4.78	0.478027	2037
457	calipers	1	4.78	0.478005	2896
458	spinach	130	4.77	0.477691	1672
459	couch	1197	4.71	0.477358	4592
460	parsley	43	4.77	0.477228	2701
461	koala	31	4.77	0.477165	2457
462	bobsleigh	12	4.77	0.477064	1768
463	highlighter	6	4.77	0.477032	1822
464	silverfish	4	4.77	0.477021	1138
465	greenhouse	112	4.76	0.476595	2400
466	blimp	53	4.76	0.476282	2092
467	tray	410	4.74	0.476178	1915
468	mandolin	24	4.76	0.476127	2779
469	spareribs	22	4.76	0.476117	2035
470	gecko	19	4.76	0.476101	2238
471	volcano	170	4.75	0.475903	1600

Continuation of Table					
category index	category name	word frequency	concreteness	FCI	# of images
472	cabbage	148	4.75	0.475786	4473
473	kettle	143	4.75	0.475760	2249
474	antenna	122	4.75	0.475648	4809
475	panda	108	4.75	0.475574	2015
476	microchip	74	4.75	0.475393	3011
477	toolbox	64	4.75	0.475340	1194
478	weasel	250	4.74	0.475328	1465
479	hairbrush	37	4.75	0.475197	1788
480	squeegee	14	4.75	0.475074	866
481	flashbulb	3	4.75	0.475016	1337
482	pretzel	102	4.74	0.474542	2161
483	sawmill	21	4.74	0.474112	1354
484	joystick	15	4.74	0.474080	2388
485	persimmon	4	4.74	0.474021	3484
486	wand	157	4.73	0.473834	1373
487	graffiti	103	4.73	0.473547	2019
488	giraffe	76	4.73	0.473404	838
489	chinchilla	27	4.72	0.472143	2365
490	sundial	23	4.72	0.472122	1947
491	beaker	22	4.72	0.472117	1500
492	honeycomb	20	4.72	0.472106	1583
493	fishnet	11	4.72	0.472058	938
494	odometer	11	4.72	0.472058	1932
495	scallion	8	4.72	0.472042	1441
496	chess	380	4.70	0.472018	4018
497	photocopier	2	4.72	0.472011	3268
498	chair	2511	4.58	0.471338	4901
499	locker	815	4.67	0.471329	1150
500	newsstand	53	4.71	0.471282	1204
501	reef	204	4.70	0.471084	3453
502	cheetah	117	4.70	0.470621	2047
503	salsa	109	4.70	0.470579	1103
504	auditorium	80	4.70	0.470425	2610
505	extinguisher	78	4.70	0.470414	1364
506	battery	633	4.67	0.470362	3227
507	dildo	68	4.70	0.470361	723
508	chestnut	65	4.70	0.470345	3170
509	manhole	38	4.70	0.470202	2923
510	gooseberry	14	4.70	0.470074	1267
511	salamander	8	4.70	0.470042	4195
512	spearmint	6	4.70	0.470032	1409
513	hotplate	5	4.70	0.470027	864
514	moth	116	4.69	0.469616	4920
515	lizard	247	4.68	0.469312	4936
516	beaver	246	4.68	0.469307	2367
517	nutmeg	36	4.69	0.469191	1360
518	cannabis	23	4.69	0.469122	1181
519	cogwheel	2	4.69	0.469011	1219
520	bolt	351	4.67	0.468864	2443
521	hut	674	4.65	0.468580	4732
522	robot	621	4.65	0.468299	3519
523	shield	418	4.66	0.468220	1933
524	chameleon	37	4.68	0.468197	3301
525	defibrillator	24	4.68	0.468127	845
526	bison	17	4.68	0.46809	3621
527	geyser	11	4.68	0.468058	2147
528	raccoon	73	4.67	0.467388	3115
529	coleslaw	63	4.67	0.467335	1847
530	thermos	57	4.67	0.467303	1477

Continuation of Table					
category index	category name	word frequency	concreteness	FCI	# of images
531	macadamia	31	4.67	0.467165	812
532	compass	207	4.66	0.467100	2848
533	ruler	162	4.66	0.466860	1740
534	helicopter	806	4.62	0.466281	3081
535	manatee	10	4.66	0.466053	1645
536	pinwheel	7	4.66	0.466037	1820
537	cherry	693	4.62	0.465681	4362
538	pickle	235	4.64	0.465248	1357
539	projector	62	4.64	0.464329	4628
540	guillotine	42	4.64	0.464223	920
541	tapioca	31	4.64	0.464165	1167
542	birdcage	24	4.64	0.464127	1320
543	coffeepot	21	4.64	0.464112	3052
544	sharpener	17	4.64	0.464090	1589
545	baguette	14	4.64	0.464074	1440
546	gearshift	11	4.64	0.464058	1702
547	pier	334	4.62	0.463774	1576
548	drain	440	4.61	0.463337	1258
549	artichoke	28	4.63	0.463149	2371
550	oscilloscope	4	4.63	0.463021	1908
551	cube	152	4.62	0.462807	2400
552	stapler	44	4.62	0.462234	1737
553	bubble	408	4.60	0.462167	3882
554	ukulele	29	4.62	0.462154	1811
555	tyrannosaurus	20	4.62	0.462106	1255
556	winterberry	1	4.62	0.462005	805
557	cauldron	24	4.61	0.461127	1028
558	cassette	93	4.60	0.460494	2583
559	meteorite	41	4.60	0.460218	990
560	urinal	39	4.60	0.460207	770
561	hazelnut	9	4.60	0.460048	907
562	rainbow	407	4.57	0.459162	2085
563	sieve	27	4.59	0.459143	1916
564	shredder	17	4.59	0.459090	1007
565	hovercraft	26	4.58	0.458138	1590
